# Supplementary material for: Predictors of Workplace Substance Reuse among Patients with Alcohol or Illegal Substance Use Disorder in the Workplace
Source: Int J Environ Res Public Health. 2022 Aug 14;19(16):10023. doi: 10.3390/ijerph191610023 (PMC9408551; doi:10.3390/ijerph191610023)
Supplement: Supplementary file 1 [file ijerph-19-10023-s001.zip › ijerph-1831333-supplementary.pdf]

Supplementary Table S1. Workplace Substance Reuse Questionnaire (Alcohol)

Has the following environment in the workplace ever made you crave a drink?		
Items	ever	never
1. Dimly illuminated		
2. Sultry air		
3. Strong smell		
4. Loud noise		
5. Frequent occupational hazards (e.g. handling corrosive substances)		
6. Frequently required to work irregular shifts (various shifts within two weeks)		
7. Often working overtime		
8. Excessive physical fatigue without adequate rest		
9. Mental stress, often needing to be released or lessened		
10. Highly repetitive work; no sense of achievement		
11. No matter how hard I try, the result is the same; I am not satisfied with the work		
12. I feel that I have to do this job in order to earn a living		
13. Bad times with coworkers or bosses		
14. The way my bosses and coworkers treat me makes me feel like I'm useless or not confident		
15. Poor, cold, or bad atmosphere among colleagues		

Supplementary Table S2. Workplace Substance Reuse Questionnaire (Illegal substance)

Has the following environment in the workplace <u>ever made you crave an illegal substance?</u>		
Items	ever	never
1. Dimly illuminated		
2. Strong smell		
3. Loud noise		
4. Frequently required to work irregular shifts (various shifts within two weeks)		
5. Often working overtime		
6. Excessive physical fatigue without adequate rest		
7. Highly repetitive work; no sense of achievement		
8. Sitting for a long time with nothing to do		
9. Bad times with coworkers or bosses		
10. The way my bosses and coworkers treat me makes me feel like I'm useless or not confident		
11. Poor, cold, or bad atmosphere among colleagues		
12. Colleagues invite me to use		
13. The company did not state that substance use is not allowed in the workplace		

Supplementary Table S3. Confirmatory factor analysis for Workplace Substance Reuse Questionnaire (Alcohol)

**Components /Items**	Factor loading	Square multiple correlation (SMC or R^2)	Composite reliability (CR)	Average Variance Extracted (AVE)
Workplace environment			0.902	0.700
Dimly illuminated	0.724	0.524		
Sultry air	0.757	0.573		
Strong smell	0.926	0.857		
Loud noise	0.920	0.846		
Workload			0.887	0.612
Often requires irregular shifts	0.709	0.503		
Often work overtime	0.867	0.752		
Excessive physical fatigue without adequate rest	0.717	0.514		
Mental stress, often needing to be refreshed or lessened	0.707	0.500		
High repetition of work; no sense of achievement	0.891	0.794		
Social interaction			0.886	0.666
Frequently occupational hazards	0.608	0.370		
Bad times with coworkers or bosses	0.841	0.707		
Bosses or coworkers treat me makes me feel like I'm useless	0.934	0.872		
Poor atmosphere among colleagues	0.846	0.716		
Other cues			0.830	0.709

I am not satisfied with the work	0.825	0.681
I feel that I have to do this job in order to earn living	0.859	0.738

Supplementary Table S4. Confirmatory factor analysis for Workplace Substance Reuse Questionnaire (Illegal substance)

**Components /Items**	Factor loading	Square multiple correlation (SMC or R^2)	Composite reliability (CR)	Average Variance Extracted (AVE)
Social interaction			0.934	0.823
Bad times with coworkers or bosses	0.835	0.697		
Bosses or coworkers treat me makes me feel like I'm useless	0.986	0.972		
Poor atmosphere among colleagues	0.894	0.799		
Workplace environment			0.846	0.649
Dimly illuminated	0.919	0.845		
Strong smell	0.744	0.554		
Loud noise	0.741	0.549		
Workload			0.815	0.598
Often requires irregular shifts	0.869	0.755		
Often work overtime	0.788	0.621		
Excessive physical fatigue without adequate rest	0.646	0.417		
Other cues			0.755	0.442
High repetition of work; no sense of achievement	0.803	0.645		
Sitting there for a long time with nothing to do	0.721	0.520		
Colleagues will invite me to use	0.500	0.250		
The company do not claim that substance is not allowed	0.595	0.354		
